# Central Hemodynamic and Thermoregulatory Responses to Food Intake as Potential Biomarkers for Eating Detection: Systematic Review

**DOI:** 10.2196/52167

**Published:** 2024-09-10

**Authors:** Lucy Chikwetu, Parker Vakili, Andrew Takais, Rabih Younes

**Affiliations:** 1 Department of Electrical and Computer Engineering Duke University Durham, NC United States; 2 Department of Computer Science Duke University Durham, NC United States

**Keywords:** eating detection, eating moment recognition, postprandial physiological responses, postprandial physiology, eating, food, consumption, postprandial, hemodynamics prandial, thermoregulation, physiological, heart rate, vital, vitals, wearable, wearables, thermoregulatory hemodynamic, biomarker, biomarkers, diet, dietary, monitoring, detect, detection, detecting, synthesis, review methods, review methodology, systematic, sensor, sensors, digital health

## Abstract

**Background:**

Diet-related diseases, such as type 2 diabetes, require strict dietary management to slow down disease progression and call for innovative management strategies. Conventional diet monitoring places a significant memory burden on patients, who may not accurately remember details of their meals and thus frequently falls short in preventing disease progression. Recent advances in sensor and computational technologies have sparked interest in developing eating detection platforms.

**Objective:**

This review investigates central hemodynamic and thermoregulatory responses as potential biomarkers for eating detection.

**Methods:**

We searched peer-reviewed literature indexed in PubMed, Web of Science, and Scopus on June 20, 2022, with no date limits. We also conducted manual searches in the same databases until April 21, 2024. We included English-language papers demonstrating the impact of eating on central hemodynamics and thermoregulation in healthy individuals. To evaluate the overall study quality and assess the risk of bias, we designed a customized tool inspired by the Cochrane assessment framework. This tool has 4 categories: high, medium, low, and very low. A total of 2 independent reviewers conducted title and abstract screening, full-text review, and study quality and risk of bias analysis. In instances of disagreement between the 2 reviewers, a third reviewer served as an adjudicator.

**Results:**

Our search retrieved 11,450 studies, and 25 met our inclusion criteria. Among the 25 included studies, 32% (8/25) were classified as high quality, 52% (13/25) as medium quality, and 16% (4/25) as low quality. Furthermore, we found no evidence of publication bias in any of the included studies. A consistent postprandial increase in heart rate, cardiac output, and stroke volume was observed in at least 95% (heart rate: 19/19, cardiac output: 18/19, stroke volume: 11/11) of the studies that investigated these variables’ responses to eating. Specifically, cardiac output increased by 9%-100%, stroke volume by 18%-41%, and heart rate by 6%-21% across these studies. These changes were statistically significant (*P*<.05). In contrast, the 8 studies that investigated postprandial thermoregulatory effects displayed grossly inconsistent results, showing wide variations in response with no clear patterns of change, indicating a high degree of variability among these studies.

**Conclusions:**

Our findings demonstrate that central hemodynamic responses, particularly heart rate, hold promise for wearable-based eating detection, as cardiac output and stroke volume cannot be measured by any currently available noninvasive medical or consumer-grade wearables.

**Trial Registration:**

PROSPERO CRD42022360600; https://www.crd.york.ac.uk/prospero/display_record.php?RecordID=360600

## Introduction

The rising incidence of diet-related diseases, such as coronary heart disease [1], and type 2 diabetes [2] has led to the emergence of an innovative research field called automated diet monitoring [3,4]. The primary objective of this field is to advance technologies that facilitate comprehensive monitoring of critical elements of food intake, such as meal timing, duration, quantity, and nutritional composition [5]. A fundamental aspect of this field is eating detection [4], which involves using technologies such as wearable devices to determine when an individual is eating. This innovative approach holds immense potential in empowering individuals to more accurately and efficiently monitor their dietary habits and effectively manage chronic diseases that require precise dietary control. By leveraging wearable technologies, eating detection enables real-time monitoring of meal timing, duration, and eating patterns, thus providing valuable insights into eating behaviors and supporting overall health and well-being.

Despite extensive research in eating detection [6-10], successful deployment of eating detection platforms remains elusive. Several challenges have hindered their widespread adoption, including the use of custom-made wearables with impractical form factors [4,11] or privacy concerns [12]. Notable examples include dental implants [11], on-body cameras [12], and wrist-worn inertial sensors [13,14] (accelerometer, gyroscope, or magnetometer), which pose significant challenges for widespread acceptance. In addition, many studies have relied on data collected in controlled laboratory or semicontrolled field settings [6,15,16], leading to algorithms that struggle to perform effectively in real-life scenarios filled with diverse activities and situations.

Furthermore, while some studies have achieved accurate detection of eating episodes [17-19], few have demonstrated the feasibility of real-time implementation [6], raising questions about their suitability for use in free-living scenarios. Real-time computation requires high-performance computing platforms with long battery life, presenting significant challenges for many proposed solutions [6]. In addition, detecting eating episodes during concurrent activities, such as walking, remains a substantial hurdle that necessitates further algorithmic improvements [7].

By the same token, most of the existing systems rely on a single sensor with limited provisions for sensor failure or suboptimal performance. In contrast, multimodal sensing, which involves using different sensors on one or more devices, can provide complementary and unique information, enhancing the performance and reliability of eating detection platforms, especially when combined with higher sampling rates [4,20].

Given the aforementioned challenges, extensive research focusing on exploring untapped sensing signals and repurposing everyday wearable devices for eating detection remains crucial. In line with this objective, our review investigates the potential of central hemodynamic and thermoregulatory responses to food intake as promising biomarkers for eating detection. Through a comprehensive analysis of the existing literature, this review aims to shed light on the role of central hemodynamics and thermoregulation in monitoring eating behavior, contributing valuable insights toward the development of effective and practical eating detection platforms.

## Methods

### Overview

This review adheres to the PRISMA (Preferred Reporting Items for Systematic Reviews and Meta-Analyses) [21] guidelines. It is registered on PROSPERO (CRD42022360600).

### Information Sources

We searched peer-reviewed literature indexed in PubMed, Web of Science, and Scopus on June 20, 2022, with no date limits. We also conducted manual searches in the same databases until April 21, 2024. Before the official search, we identified a key study [22] and decided to augment our search by retrieving officially provided similar studies from PubMed and Web of Science.

### Search Strategy

This review examines central hemodynamic and thermoregulatory responses to food intake as potential biomarkers for wearable-based eating detection. To capture relevant studies, our search used keywords such as “hemodynamic,” “haemodynamic*,” “thermoregulat*,” “temperature regulation,” “body heat,” “body temperature,” “skin temperature,” “eating,” “meal*,” “ingest*,” “intak*,” “postprandial,” and “post-prandial.” We excluded all animal studies from our search. While our search strategy did not impose language restrictions on retrieved papers, we excluded 10 non–English-language papers published in Czech, French, German, and Japanese. For a comprehensive overview of our search strategy across all databases, please refer to Multimedia Appendix 1.

### Eligibility Criteria

All included studies were peer-reviewed journal and conference papers published before April 21, 2024. Eligible studies had to demonstrate the impact of eating on central hemodynamics and thermoregulation, assessing metrics such as changes in heart rate, blood pressure, cardiac output, core or skin temperature, or stroke volume in healthy individuals.

We excluded non-English papers, animal research studies, opinion pieces, letters, studies not specifically focusing on central hemodynamics (eg, those focusing on renal dynamics), studies using indirect calorimetry to measure postprandial thermogenesis, studies testing drug effects, studies focusing on water ingestion, long-term effects of food, and studies primarily examining central hemodynamic and thermoregulatory responses to food intake during or after exercise.

### Screening and Selection

All retrieved studies were exported to Covidence [23], which automatically identified and removed 1419 duplicates. In total, 2 reviewers independently conducted the title and abstract screening and the full-text review. In cases of disagreement between the 2 reviewers, a third reviewer acted as an adjudicator.

### Data Extraction and Synthesis

A total of 2 reviewers independently conducted data extraction, study quality assessment, and risk-of-bias assessment for each included study. Any conflicts or discrepancies were resolved by an adjudicator. In addition, if any of the included studies referenced other studies that met our eligibility criteria, we also extracted relevant information from those referenced studies. We extracted 8 study characteristics from the included studies (Multimedia Appendix 2).

Due to the heterogeneity in the outcomes and designs of the included studies, we used a narrative data synthesis approach. In our characteristic tables (Multimedia Appendix 2), we summarized key findings from each study, identifying common themes, patterns, and differences. This approach allowed us to integrate both quantitative and qualitative data, thus providing a comprehensive overview of the current landscape. Finally, all graphs were generated using ggplot2 in R (version 4.0.2; R Foundation for Statistical Computing).

## Results

**Figure 1:**
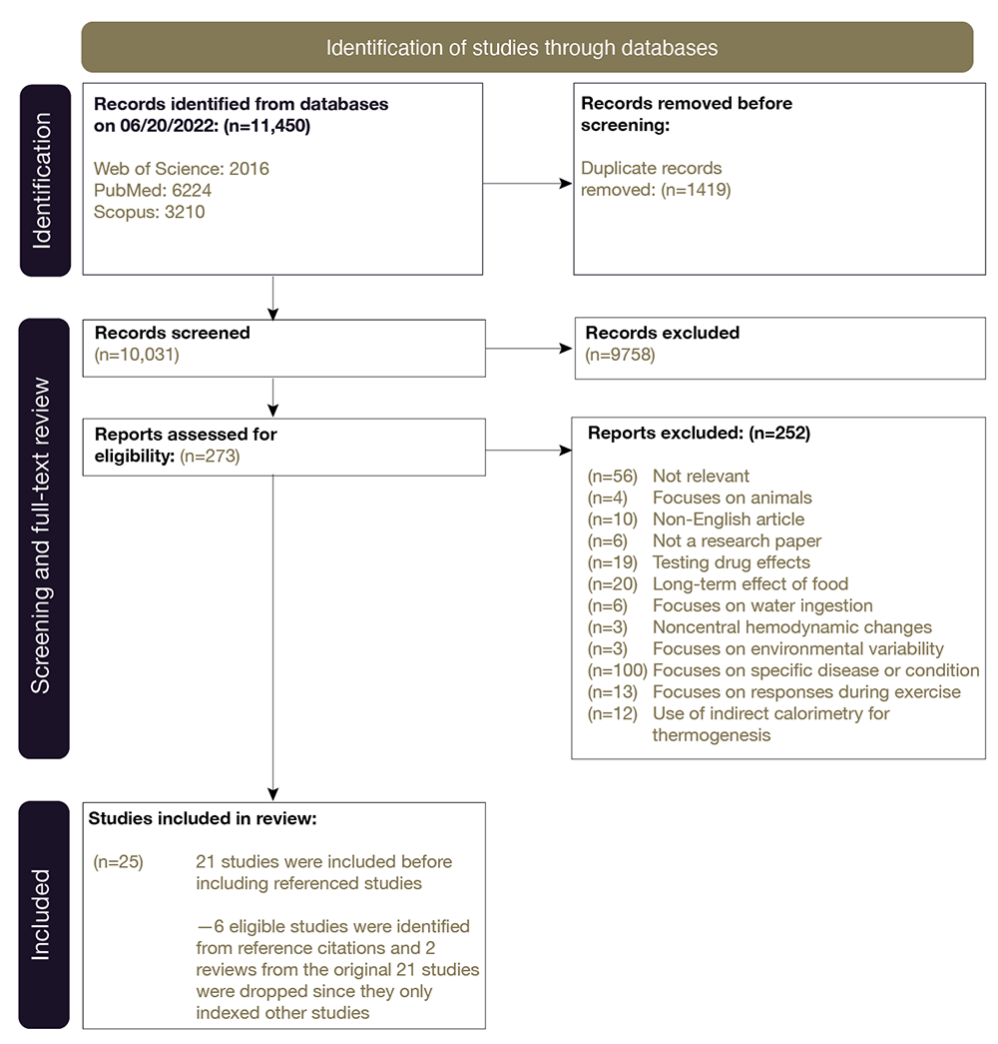
PRISMA (Preferred Reporting Items for Systematic Reviews and Meta-Analyses) diagram illustrating the article selection process.

Our search retrieved 11,450 studies (1419 duplicates), resulting in 10,031 studies being screened (Figure 1). Following the title and abstract screening, 273 studies progressed to the full-text review. Out of these, 21 studies met the eligibility criteria. In addition, we conducted a nested search within the references of the 21 studies and identified 6 additional relevant studies. We excluded 2 studies from the original 21 as they were review papers referencing other studies already included in our analysis. Finally, we extracted data from the included 25 studies and proceeded to analyze them.

To assess overall study quality and risk of bias, we developed a custom tool comprising 4 categories: high, medium, low, and very low (Multimedia Appendix 3). This assessment tool was based on the Cochrane assessment framework [24]. In total, 2 reviewers independently evaluated the study quality and risk of bias for each study, with an adjudicator resolving any conflicts. Among the 25 included studies, 32% (8/25) were classified as high quality, 52% (13/25) as medium quality, and 16% (4/25) as low quality (Multimedia Appendix 3). Furthermore, we found no evidence of publication bias in any of the included studies.

Of the included studies, 14 physiological responses were recorded. Multimedia Appendix 4 presents a breakdown of the number of studies reporting each postprandial physiological response. The number of participants per study ranged from 4 to 104, with a mean of 17 (SD 19). Out of the 416 participants in the 25 included studies, 230 (55.3%) were male, 180 (44.3%) were female, and the remaining 6 (1.4%) had unknown sex. The age of the participants in the included studies ranged from 18 to 69 years, and all participants were healthy. The sessions varied in duration, spanning from 10 minutes to 8 hours, and the provided food items included cake, cheese, filet mignon, and boiled eggs, among other foods.

Our findings overwhelmingly demonstrate a significant increase in heart rate after eating; 19 included studies investigated heart rate, and all 19 studies showed a statistically significant (*P*<.05) rise ranging from 6% to 21% (Multimedia Appendix 2). Furthermore, the postprandial effects of heart rate were found to be generally similar in both supine and erect positions (Multimedia Appendix 2). In addition, there was strong evidence supporting an increase in cardiac output after food ingestion, with 18 of the 19 studies on cardiac output showing a statistically significant (*P*<.05) postprandial increase ranging from 9% to 100%, and only 1 study showing a statistically insignificant (*P*>.05) response for a high-fat liquid meal consisting of emulsified peanut oil (Multimedia Appendix 2). Similarly, data from 11 studies revealed strong evidence supporting a postprandial increase in stroke volume, ranging from 18% to 41% (Multimedia Appendix 2). These results collectively highlight the consistent and significant cardiovascular responses associated with food intake.

Out of the 25 included studies, 16 (64%) investigated the response of blood pressure to food ingestion; however, the results were inconclusive (Multimedia Appendix 2). Among these studies, 7 observed a postprandial increase in systolic blood pressure, while 4 found a statistically insignificant (*P*>.05) response, and 1 study even reported a postprandial decrease. Similarly, 8 studies found a postprandial decrease in diastolic blood pressure, while 3 studies found a statistically insignificant (*P*>.05) response, and 1 study observed a postprandial increase. Regarding mean blood pressure, the findings indicated either inconsistent postprandial behavior or a statistically insignificant (*P*>.05) response (Multimedia Appendix 2).

A total of 2 studies investigated the response of vascular resistance to food ingestion; one study focused solely on systemic vascular resistance, while the other study researched both systemic and mesenteric vascular resistance (Multimedia Appendix 2). Both studies observed a statistically significant (*P*<.05) postprandial decrease in vascular resistance.

In addition, 6 studies examined the response of blood flow in the hand, calf, and superior mesenteric artery to food ingestion (Multimedia Appendix 2). However, the postprandial effects of blood flow in the hand or calf were unclear. A total of 2 studies indicated a statistically significant (*P*<.05) postprandial increase in hand and calf blood flow, while 3 studies found a statistically insignificant (*P*>.05) change (Multimedia Appendix 2). Furthermore, 5 studies explored the response of oxygen uptake to food intake. In total, 3 of these studies observed a postprandial increase in oxygen uptake (more details in Multimedia Appendix 2). In addition, 1 study showed an inconsistent response, and another reported a statistically insignificant change (*P*>.05). Finally, the postprandial effects of skin or core temperature were found to be grossly inconsistent (Multimedia Appendix 2). A total of 5 studies reported inconsistent responses of skin or core temperature to food ingestion, 2 studies observed a postprandial increase, and 1 study found a statistically insignificant (*P*>.05) response (Multimedia Appendix 2).

## Discussion

The primary objective of this systematic review was to investigate central hemodynamic and thermoregulatory responses to food intake as potential biomarkers for detecting eating events. Among the 25 studies included in this review, at least 95% (heart rate: 19/19, cardiac output: 18/19, stroke volume: 11/11) of those investigating the response of heart rate, cardiac output, and stroke volume to food ingestion reported consistent, statistically significant (*P*<.05) elevations in these variables. In contrast, the postprandial thermoregulatory effects were markedly inconsistent across the 8 studies that investigated them.

Our findings provide valuable insights into the physiological changes that occur after food consumption and shed light on potential biomarkers for detecting eating events. They consistently demonstrate significant cardiovascular responses associated with food intake (Multimedia Appendix 2). Specifically, heart rate showed a significant increase after eating, as evidenced by 19 studies, with the rise ranging from 6% to 21% (Multimedia Appendix 4 and Textbox 1). Similarly, cardiac output and stroke volume exhibited a robust postprandial increase, with 18 studies on cardiac output showing a significant rise ranging from 11% to 100%, and 11 studies revealing a postprandial increase in stroke volume, ranging from 18% to 41% (Multimedia Appendix 4 and Textbox 1). These findings suggest that heart rate, cardiac output, and stroke volume may serve as reliable biomarkers for detecting eating events. However, it is worth mentioning that 1 study reported a statistically insignificant (*P*>.05) cardiac output response to a high-fat liquid meal [25], while 9 studies reported different postprandial effects depending on the composition of the consumed food, underscoring the potential influence of dietary composition on cardiovascular reactions. Consequently, it is worth exploring whether eating detection using cardiovascular responses, particularly heart rate, could be enhanced to sense macronutrients in the ingested food. A particular area of focus could be carbohydrate-aware eating detection platforms, which would have the ability to classify the amount of carbohydrates in the food a person has just eaten into 2 classes that are high-carbohydrate and low-carbohydrate. We anticipate this to be feasible since several studies have demonstrated that postprandial heart rate changes within the first hour of eating correlate with the carbohydrate content in the food an individual has just consumed [25-28]. Accordingly, individuals could receive alerts when their meals are carbohydrate-rich, thus empowering them with more information about the foods they consume. In addition, decision support systems for diabetes management could use the inferred postprandial carbohydrate information to improve patient outcomes by personalizing patient recommendations based on their diet information.

**Postprandial percentage increase range for cardiac output, stroke volume, and heart rate.**

**Postprandial percentage increase range**

- Cardiac output: 9%-100%
- Stroke volume: 18%-41%
- Heart rate: 6%-21%

Currently, there are no noninvasive consumer or medical-grade wearables capable of measuring cardiac output and stroke volume. As a result, heart rate remains the only signal that could feasibly be used in everyday eating detection platforms. Studies using heart rate for eating detection are already underway, but they have primarily focused on animal studies so far [29,30]. A notable study conducted in humans used consumer smartwatches and successfully detected eating events using heart rate with an accuracy of 98.6% [31]. However, the sensitivity, specificity, and *F*_1_-scores were very low, with an *F*_1_-score as low as 2% in one of the experiments [31]. We anticipate that using high-resolution data and conducting initial experiments in stationary situations without vigorous physical activity could facilitate a better understanding of heart rate–based eating detection systems.

Conversely, the findings concerning blood pressure responses were inconclusive. Of the 25 studies included, 16 (64%) investigated blood pressure changes following food ingestion; however, the results were inconsistent for systolic, diastolic, and mean blood pressure changes (Multimedia Appendix 2). The lack of consensus on blood pressure responses may limit its potential as a reliable biomarker for eating detection using wearable devices.

Vascular resistance and blood flow were also examined in a subset of the included studies (n=2 and n=6, respectively). Interestingly, vascular resistance showed a consistent postprandial decrease in both studies that investigated this parameter (Multimedia Appendix 2). On the other hand, the effects of postprandial blood flow in the hand, calf, and superior mesenteric artery were inconclusive, with some studies indicating postprandial effects, while others found no significant changes (Multimedia Appendix 2). Consequently, the use of vascular resistance and blood flow as biomarkers for eating detection may require further investigation and validation.

Furthermore, oxygen uptake responses after food intake were explored in 5 studies (Multimedia Appendix 2). While 3 studies showed a postprandial increase in oxygen uptake, 1 study reported inconsistent results, and another found no significant change. These mixed findings suggest that oxygen uptake may have limited use as a standalone biomarker for wearable-based eating detection.

Finally, postprandial temperature effects were found to be grossly inconsistent (Multimedia Appendix 2). While many studies have focused on determining the thermic effect of food [32-34] through calorimetry, very little is known about the skin or core temperature response to eating, and this review aimed to bridge that gap. Among the included studies, 2 reported a postprandial increase in temperature, while 5 studies revealed inconsistent responses, and 1 study found no significant change (Multimedia Appendix 2). The inconsistency in temperature responses warrants caution when considering temperature-based biomarkers for eating detection. In addition, temperature can be easily affected by environmental factors, and there is often a time lag before individuals elicit any temperature change in response to any physiological or environmental factors.

While our systematic review provides valuable insights, there are several limitations that should be acknowledged. First, the available literature on central hemodynamic and thermoregulatory responses to food intake might be limited, potentially leading to a restricted pool of studies to learn from. Sample sizes and study methodologies varied significantly, and this heterogeneity could have affected the ability to draw definitive conclusions for many variables. Furthermore, the majority of the studies focused on heart rate, cardiac output, and stroke volume, leaving other potential biomarkers underexplored. The inconsistency in the postprandial effects on blood pressure, blood flow, oxygen uptake, and temperature further highlights the need for more comprehensive research in these areas. In addition, it is important to note that all included studies were based on healthy participants, so the results from this review might not be fully extensible to the broader population, and their applicability to individuals with specific health conditions remains uncertain.

Out of the 25 included studies, 19 (76%) were conducted before 2000 using contemporary devices, which might have compromised the results’ quality compared with more recent studies that may have benefitted from the advances in modern technology. Recent advancements in precision, data collection, and analytical tools could significantly improve the accuracy and reliability of these findings. All studies included in this review had the goal of wanting to understand the postprandial effects of the concerned signals, and they were measuring changes in a discrete fashion as opposed to a continuous fashion. Furthermore, the quality assessment of the included studies indicated that 17 (68%) of the studies were categorized as medium or low quality, mostly because they did not provide information on the devices used or the frequency at which data was collected. While efforts were made to minimize bias during the review process, the quality of data reported in the primary studies could influence the overall robustness of our findings.

Overall, the insights gained from this systematic review provide a strong foundation for future research and the development of wearable technologies aimed at enhancing eating detection and monitoring. Central hemodynamic responses, particularly heart rate, offer promising prospects for wearable-based eating detection. As other identified biomarkers, such as cardiac output and stroke volume cannot be measured by any currently available noninvasive medical or consumer-grade wearable, heart rate remains a valuable signal for this purpose.

Given the consistent postprandial heart rate effects in both supine and erect positions, heart rate–based eating detection technologies have the potential to revolutionize assisted living care, particularly in enhancing monitoring and support for bedridden individuals. For instance, this technology could track and verify that patients are receiving meals at prescribed times, providing reassurance to doctors and family members that dietary guidelines are being followed and proper care is being administered. Despite its potential, the complexity of the heart rate signal, influenced by factors such as physical exercise [35] and stress responses [36], may complicate its ability to detect eating events. To accurately identify eating episodes, algorithms must account for these variables and differentiate between heart rate changes due to eating and other activities. Integrating heart rate data with additional sensors, such as electrodermal activity (EDA) sensors, which measure skin conductance and emotional states [37,38], and inertial measurement units (IMUs) sensors, such as accelerometers and gyroscopes, which monitor physical activity or hand movements, could enhance algorithm performance. This multimodal approach increases context awareness, allowing for clearer distinctions between physiological changes from eating and other activities, such as exercise or emotional states. By reducing false positives, such systems can become more sensitive and specific to changes associated with eating. It is also important to note, however, that many signals, including heart rate, EDA, and IMUs, carry the risk of reidentification [39]. Therefore, any systems using these signals should be properly secured to mitigate this privacy concern.

In conclusion, central hemodynamic responses, particularly heart rate, show promise for wearable-based eating detection. Future studies could aim for larger sample sizes, standardized protocols, and well-controlled experimental conditions to enhance the generalizability and reliability of the results. In addition, investigating how these findings apply to diverse populations and individuals with specific health conditions is crucial for broader application and impact.

## Appendix

Multimedia Appendix 1 Search strategy.

Multimedia Appendix 2 Characteristics of included studies.

Multimedia Appendix 3 Study quality and publication bias assessment.

Multimedia Appendix 4 Postprandial physiological responses and the number of studies reporting data for each category.

Multimedia Appendix 5 PRISMA Checklist.

## Abbreviations

EDA: electrodermal activity
IMU: inertial measurement units
PRISMA: Preferred Reporting Items for Systematic Reviews and Meta-Analyses

## Data Availability

All data generated in this study are available in the appendices. The search strategy can be found in Multimedia Appendix 1, and the list of extracted study characteristics for each included study is in Multimedia Appendix 2. In addition, the risk-of-bias and study quality assessments are provided in Multimedia Appendix 3, and the postprandial physiological responses, along with the number of studies reporting data for each category, are in Multimedia Appendix 4.
