# Supplementary material for: Central Hemodynamic and Thermoregulatory Responses to Food Intake as Potential Biomarkers for Eating Detection: Systematic Review
Source: Interact J Med Res. 2024 Sep 10;13:e52167. doi: 10.2196/52167 (PMC11422732; doi:10.2196/52167)
Supplement: Multimedia Appendix 1 [file ijmr_v13i1e52167_app1.pdf]

Multimedia Appendix 1

Search Strategy

We performed the search on **20 June 2022** and the results are shown below.

	Web of Science	Scopus	PubMed	relPubMed	relWebOfSci	Total	After dedup
Search	1,919	3,210	6,182	42	97	11,450	10,056

Web of Science
((hemodynamic OR haemodynamic*) OR (thermoregulat* OR "temperature regulation" OR "body heat" OR "body temperature" OR "skin temperature")) (Topic) AND (eating OR meal* OR ingest* OR intak* OR postprandial OR "post-prandial") (Topic) NOT (animal* OR rat* OR mouse OR mice OR chicken OR dog* OR fish OR pig* OR mosquito OR Haematophagy) (Topic)

Scopus
(TITLE-ABS-KEY (((hemodynamic OR haemodynamic*) OR (thermoregulat* OR "temperature regulation" OR "body heat" OR "body temperature" OR "skin temperature"))) AND TITLE-ABS-KEY ((eating OR meal* OR ingest* OR intak* OR postprandial OR "post-prandial")) AND NOT TITLE-ABS-KEY ((animal* OR rat* OR mouse OR mice OR pig* OR fish OR dog* OR chicken OR mosquito OR haematophagy)))

PubMed
((hemodynamic[MeSH Major Topic]) OR (thermoregulation[MeSH Terms])) AND (("eating"[MeSH Major Topic]) OR meal* OR intak* OR ingest* OR postprandial OR "post-prandial") NOT (animal* OR rat* OR mouse OR mice OR pig* OR chicken* OR dog* OR fish OR mosquito OR Haematophagy)

Key Article
Central haemodynamic changes after a meal by Kelbaek et al.

relPubMed ⇒ related papers to the key article (search for related papers was performed in PubMed)
relWebOfSci ⇒ related papers to the key article (search for related papers was performed in Web of Science)
