# Supplementary material for: Central Hemodynamic and Thermoregulatory Responses to Food Intake as Potential Biomarkers for Eating Detection: Systematic Review
Source: Interact J Med Res. 2024 Sep 10;13:e52167. doi: 10.2196/52167 (PMC11422732; doi:10.2196/52167)
Supplement: Multimedia Appendix 2 [file ijmr_v13i1e52167_app2.pdf]

Multimedia Appendix 2

Characteristics of included studies

Study	Subjects	Mean Age +/- SD (Age Range)	Sessions	Foods	Postprandial Physiological Changes
[1]	24 male	≈27.8 ± 7 yrs	1 (4.5 hours)	*Carbohydrate- rich meal (8 participants)*<br>● apple juice, apple sauce, ice-cream, butter cake, maple syrup<br><br>*Protein-rich meal (8 participants)*<br>● filet mignon<br><br>*Control (8 participants)*<br>● fasting	**Heart Rate:** increase [protein: 21%, carbohydrate: 7%]<br><br>**Systolic Blood Pressure:** increase [protein: 9%, carbohydrate: 9%]<br><br>**Diastolic Blood Pressure:** insignificant change<br><br>**Cardiac Output:** increase [protein: 46%, carbohydrate: 34%]<br><br>**Oxygen Uptake:** increase [protein: 31%, carbohydrate: insignificant change]
[2]	8 male	25 ± 3 yrs	2 (8 hours)	*Standard meal (all participants on a random day)*<br>● 1 boiled egg, 2 bread rolls, 3 tsp margarine, 40g ham, 40g Edamer cheese, and 230 ml orange juice<br><br>*Control (all participants on the other day)*<br>● placebo + water	**Heart Rate:** increase [supine:11%, erect:12%]<br><br>**Systolic Blood Pressure:** insignificant change<br><br>**Diastolic Blood Pressure:** decrease [supine:16%, erect:14%]
[3]	8 male	25 ± 3 yrs	2 (5 hours)	*Standard meal (all participants on a random day)*<br>● 1 boiled egg, 2 bread rolls, 3 tsp margarine, 40g ham, 40g cheese, 230 ml juice<br><br>*Control (all participants on the other day)*<br>● fasting	**Heart Rate:** increase [13%]<br><br>**Systolic Blood Pressure:** increase [4%]<br><br>**Diastolic Blood Pressure:** decrease [18%]
[4]	12 male	27 ± 2 yrs	2 (4 hours)	*Standard meal (all participants on a random day)*<br>● 1 boiled egg, 2 bread rolls, 3 tsp margarine, 40g ham, 40g cheese, 230 ml	**Heart Rate:** increase [+8.1 bpm]<br><br>**Systolic Blood Pressure:** increase [+4 mmHg]

Study	Subjects	Mean Age +/- SD (Age Range)	Sessions	Foods	Postprandial Physiological Changes
				juice<br><br>*Control (all participants on the other day)*<br>● placebo + water	**Diastolic Blood Pressure:** decrease [-8 mmHg]<br><br>**Cardiac Output:** increase [+1.4 l/min]
[5]	11 male<br>12 female	25.6 ± 4.5 yrs	1 (110 minutes)	*Standard meal (all participants)*<br>● 300 g rice pudding	**Heart Rate:** increase [7%]<br>**Systolic Blood Pressure:** insignificant change<br><br>**Diastolic Blood Pressure:** decrease [12%]<br><br>**Cardiac Output (left ventricular):** increase [28%]<br><br>**Stroke Volume (left ventricular):** increase [20%]
[6]	13 male<br>7 female	30 ± 9 yrs<br>(18-52 yrs)	1(≈ 1 hour)	*Standard Meal (all 20 participants)*<br>● 300 ml double heavy cream, 89 g maltose syrup, natural flavoring<br><br>*Control (10 random participants)*<br>● water	**Heart Rate:** increase [approx. 13%]<br><br>**Mean Blood Pressure:** insignificant change<br><br>**Stroke Volume:** increase [approx. 8%]<br><br>**Cardiac Output:** increase [20%]<br><br>**Systemic Vascular Resistance:** decrease [-20%]
[7]	4 male<br>5 female	24 yrs<br>(21-33 yrs)	5 (4 hours)	*Carbohydrate-rich meal (all participants)*<br>● maltodextrin<br><br>*Fat-rich meal (all participants)*<br>● emulsified peanut oil<br><br>*Protein-rich meal (all participants)*<br>● Soluble milk whey protein<br><br>*Balanced Diet (all participants)*<br>● Fortisip Energy Plus (Cow and Gate)<br><br>*Control (all participants)*<br>● water	**Heart Rate:** increase<br><br>**Mean Blood Pressure:** no significant change<br><br>**Stroke Volume:** increase<br><br>**Cardiac Output:** increase (balanced diet: 44%, carbohydrate: 28%, protein: 22%, but NOT with the fat diet)
[8]	8 male	20-27 yrs	4 (2 hours)	*Carbohydrate-rich meal (all participants)*<br>● Unspecified liquid meal	**Heart Rate:** increase [11-16%]<br><br>**Cardiac Output (left

Study	Subjects	Mean Age +/- SD (Age Range)	Sessions	Foods	Postprandial Physiological Changes
				Fat-rich meal (all participants)<br>● Unspecified liquid meal<br>*Protein-rich meal (all participants)*<br>● Unspecified liquid meal<br><br>*Control (all participants)*<br>● water	**ventricular):** increase [35-40%]<br><br>**Stroke Volume (left ventricular):** increase [18-22%]<br><br>**Forearm Blood Flow:** increase [carbohydrate and protein]
[9]	13 male<br>6 female	21-69 yrs	1 (5 hours)	*800 Kcal unspecified lunch with 100-200 ml water*<br>*Glucose*<br>● 75 g of glucose dissolved in flavored water	**Heart Rate:** increase [lunch: 19%, glucose: 5%]<br>**Stroke Volume:** increase [lunch: 18%, glucose: insignificant change]<br>**Cardiac Output:** increase [lunch: 37%, glucose: 12%]
[10]	7 male<br>1 female	19-31 years	2(≈45 minutes)	*Standard meal (all participants)*<br>● 280 g minced beef, 300 g potatoes, 50 g peas, 40 g sliced cucumber, sauce, 150 g stewed apples with 30 g cream, and 400 g low-fat milk<br><br>*Control*<br>● unspecified	**Heart Rate:** increase [17%]<br><br>**Cardiac Output:** increase [62%]<br><br>**Stroke Volume:** increase [41%]<br><br>**Blood Pressure:** insignificant change
[11]	4 male		≥180 minutes	*Amino Acid at 24 ℃*<br>● Glycine<br>● Phenylalanine<br>● Histidine<br>● Glutamic Acid<br>● Tyrosine<br>● Leucine<br>● Methionine<br><br>*Amino Acid I at 18 ℃ or 30 ℃*<br>● glycine	**Hand Skin Temperature at 24℃:**<br>● increase [glycine: 3.5-7.0℃]<br>● undefined increase [histidine, phenylalanine]<br>● No effect [glutamic acid, tyrosine, leucine, methionine]<br><br>**Hand Blood Flow at 24℃:**<br>● average increase [2.5-7X]<br>● undefined increase [histidine, phenylalanine]<br>● No effect [glutamic acid, tyrosine, leucine, methionine]<br><br>**Hand Skin Temperature at 18℃ or 30℃:**<br>● no significant change [glycine]<br><br>**Hand Blood Flow at 18℃ or 30℃:**

Study	Subjects	Mean Age +/- SD (Age Range)	Sessions	Foods	Postprandial Physiological Changes
					• no significant change [glycine]
[12]	36 male<br>68 female	18-25 years	1 (3 hours 20 minutes)	*Standard meal (all participants)*<br>• unspecified	**Mean, Proximal, and Distal Skin Temperature:**<br>• increase [males & females]<br><br>**Supraclavicular Skin Temperature:**<br>• increase [females]<br>• no significant change [males]
[13]	7 male<br>7 female	30 ± 2 years	1-2 (1 hour)	*Standard meal (all participants)*<br>• 800kcal consisting of 35 g fat, 35 g protein, and 90 g carbohydrate<br><br>*Control (8 participants)*<br>• fasting	**Heart Rate:** increase [18.5%]<br><br>**Systolic Blood Pressure:** no significant change<br><br>**Diastolic Blood Pressure:** decrease<br><br>**Cardiac Output:** increase [27%]<br><br>**Superior Mesenteric Blood Flow:** increase [84%]<br><br>**Calf Blood Flow:** increase<br><br>**Systemic Vascular Resistance:** decrease<br><br>**Superior Mesenteric Vascular Resistance:** decrease
[14]	9 male<br>4 female	23 ± 0.2 years	3 (> 10 minutes)	*Food (all participants)*<br>• Two pieces of 20 g maple-flavored Calorie Mate (total caloric content of 200 kcal, with 20.1 g of carbohydrates, 11.3 g of fat, and 4 g of protein)<br><br>*Gum (all participants)*<br><br>*Water (all participants)*	**Systolic Blood Pressure:** increase (food and gum)<br><br>**Diastolic Blood Pressure:** increase (food and gum)<br><br>**Cardiac Output:** increase (food and gum)<br><br>**Stroke Volume:** increase (food and gum)
[15]	7 male<br>4 female	21-32 years	≥80 minutes	*Food (all participants)*<br>• CH-19 sweet pepper<br>• California Wonder pepper	**Core Temperature:**<br>• increase[CH-19 sweet pepper]<br>• decrease [California Wonder]<br><br>**Wrist Temperature**<br>• increase [CH-19 sweet

Study	Subjects	Mean Age +/- SD (Age Range)	Sessions	Foods	Postprandial Physiological Changes
					pepper and California Wonder]<br><br>**Neck Temperature**<br>● increase [CH-19 sweet pepper]<br>● decrease [California Wonder]<br><br>**Oxygen Consumption**<br>● increase [CH-19 sweet pepper]<br>● decrease [California Wonder]
[16]	21 male<br>3 female	18-24 years	1 (7-8 hours)	*500-800 calorie meals (all participants)*<br>● Unspecified composition	**Finger Temperature:** inconsistent<br>**Toe Temperature:** inconsistent
[17]	9 male<br>8 female	29 ± 2 years	>180 minutes	*Liquid Meal (all participants)*<br>● Ensure (Abbot Laboratories, Columbus, OH)	**Heart Rate:** increase (6%)<br>**Cardiac Output:** increase (9%)
[18]	2 male<br>3 female	21-22 years	4 (2 hours)	*Liquid Meals (all participants)*<br>● Carbohydrate<br>● Protein<br>● Fat<br><br>*Control (all participants)*<br>● Water	**Heart Rate:** increase [20%]<br>**Stroke Volume:** increase<br>**Cardiac Output:** increase [38%]
[19]	2 male<br>5 female	22-50 years	2 or 3 (≥90 minutes)	*Standard-meal (all participants)*<br>● 120-150 g bread with butter and spread (cheese, mackerel and liver paste)<br>● 150-200 ml skimmed milk	**Cardiac Output:** increase (11-63%)
[20]	7 male<br>1 female		>4 hours	*Protein meal (all participants)*<br>● lean meat, cottage cheese, egg white and gelatine<br><br>*Carbohydrate meal (all participants)*<br>● vegetables, sweetened stewed and raw fruit, and sweetened fruit juice	**Heart Rate:** increase [carbohydrate: 9 bpm, protein: 11 bpm]<br><br>**Systolic Blood Pressure:** increase [carbohydrate and protein]<br><br>**Diastolic Blood Pressure:** no significant change [carbohydrate and protein]<br><br>**Oxygen Consumption:** increase [carbohydrate and protein]

Study	Subjects	Mean Age +/- SD (Age Range)	Sessions	Foods	Postprandial Physiological Changes
					**Forearm, Leg or Hand Blood Flow:** inconsistent [carbohydrate and protein]<br><br>**Cardiac Output:** increases [carbohydrate and protein]
[21]	5 male<br>3 female	22.3 +/- 4.6 years (16-31 years)	4-5 (180 minutes)	*Standard meals (all participants)*<br>● unspecified	**Heart Rate:** increase<br><br>**Cardiac Output:** increase<br><br>**Systolic Blood Pressure:** insignificant change<br><br>**Diastolic Blood Pressure:** insignificant change<br><br>**Stroke Volume:** increase<br><br>**Total Body VO2:** increase
[22]	11 male<br>6 female	19-31 years	≥5 hours	*Food (all participants)*<br>Standard meal (8 males)<br>● 44 g protein, 95 g carbohydrate, and 30 g fat<br>Participant Chosen Meal from Cafeteria (6 females and 3 males)<br>● unspecified<br><br>*Sham meal (6 females and 3 males)*<br>● unspecified	**Heart Rate:** increase [8-15%]<br><br>**Cardiac Output:** increase [36%]<br><br>**Systolic Blood Pressure:** increase [3-5%]<br><br>**Diastolic Blood Pressure:** decrease [3-11%]<br><br>**Stroke Volume:** increase [20%]
[23]	6		>4 hours	*Light Meal*<br>● sandwich (bread and meat) and a cup of coffee (containing sugar and cream<br><br>*Heavy Meal*<br>● bread, meat, sweet chocolate, and 150 ml of milk	**Heart Rate:** increase<br><br>**Cardiac Output:** increase [light meal: 80%, heavy meal: 100%]<br><br>**Systolic Blood Pressure:** increase<br><br>**Diastolic Blood Pressure:** decrease<br><br>**Oxygen Consumption:** increase
[24]	35 female	25.9 +/- 4.8 years, 19-39 years	1	*Unspecified Food (all participants)*<br>● normal-sized lunch (12 participants)<br>● oversized lunch [40% more energy] (11 participants)<br>● undersized lunch [40%	**Heart Rate:** increase [mean: 10%]<br><br>**Systolic Blood Pressure:** decrease [1%]<br><br>**Diastolic Blood Pressure:** decrease [6%]

Study	Subjects	Mean Age +/- SD (Age Range)	Sessions	Foods	Postprandial Physiological Changes
				less energy] (12 participants)	
[25]	2 male<br>2 female	21-25	4 (>2 hours)	*Food (all participants)*<br>small meal<br>● ham, brown bread, margarine, potato salad, milk with 2.5% fat<br><br>large meal<br>● same composition as small meal, but 2.5 times more energy	**Heart Rate:** increase<br><br>**Stroke Volume:** increase<br><br>**Cardiac Output:** increase [small meal 26%, large meal 38%]<br><br>**Mean Arterial Blood Pressure:** inconsistent

References

1. Dagenais GR, Oriol A, McGregor M. Hemodynamic effects of carbohydrate and protein meals in man: rest and exercise. J Appl Physiol 1966 Jul;21(4):1157–1162. doi: 10.1152/jappl.1966.21.4.1157
2. De Mey C, Enterling D, Brendel E, Meineke I. Postprandial changes in supine and erect heart rate, systemic blood pressure and plasma noradrenaline and renin activity in normal subjects. Eur J Clin Pharmacol 1987 Sep;32(5):471–476. doi: 10.1007/BF00637672
3. De Mey C, Hansen-Schmidt S, Enterling D. Postprandial haemodynamic changes: a source of bias in cardiovascular research affected by its own methodological bias. Cardiovasc Res 1988 Oct 1;22(10):703–707. doi: 10.1093/cvr/22.10.703
4. De Mey C, Hansen-Schmidt S, Enterling D, Meineke I. Time course and nature of postprandial haemodynamic changes in normal man. Clin Physiol 1989 Feb;9(1):77–87. doi: 10.1111/j.1475-097X.1989.tb00958.x
5. Dencker M, Björgell O, Hlebowicz J. Effect of Food Intake on Commonly Used Pulsed Doppler and Tissue Doppler Measurements: Food Intake and Doppler Measurements. Echocardiography 2011 Sep;28(8):843–847. doi: 10.1111/j.1540-8175.2011.01451.x
6. Hauser JA, Muthurangu V, Steeden JA, Taylor AM, Jones A. Comprehensive assessment of the global and regional vascular responses to food ingestion in humans using novel rapid MRI. Am J Physiol-Regul Integr Comp Physiol 2016 Mar 15;310(6):R541–R545. doi: 10.1152/ajpregu.00454.2015
7. Hawley SK, Channer KS. Relative effects of fat-, carbohydrate- and protein-containing liquid diets on cardiac output in healthy adult subjects. Clin Sci 1992 Oct 1;83(4):483–487. doi: 10.1042/cs0830483
8. Høst U, Kelbaek H, Rasmusen H, Court-Payen M, Christensen NJ, Pedersen-Bjergaard U, Lorenzen T. Haemodynamic Effects of Eating: The Role of Meal Composition. Clin Sci 1996 Apr 1;90(4):269–276. doi: 10.1042/cs0900269
9. Ishimine M, Takamoto T, Nitta M, Marumo F, Murakami K, Takasu N. Postprandial Hemodynamic Changes Evaluated by a Doppler Echocardiographic Method. Jpn Heart J 1994;35(1):35–42. doi: 10.1536/ihj.35.35
10. Kelbaek H, Munck O, Christensen NJ, Godtfredsen J. Central haemodynamic changes after a meal. Heart 1989 Jun 1;61(6):506–509. doi: 10.1136/hrt.61.6.506
11. Macht MB, Pillion EL. Changes in skin temperature and blood flow of hand following ingestion of certain amino acids. Fed Proc 1948 Mar;7(1 Pt):75. PMID:18938593
12. Martinez-Tellez B, Ortiz-Alvarez L, Sanchez-Delgado G, Xu H, Acosta FM, Merchan-Ramirez E, Muñoz-Hernandez V, Martinez-Avila WD, Contreras-Gomez MA, Gil A, Labayen I, Ruiz JR. Skin temperature response to a liquid meal intake is different in men than in women. Clin Nutr 2019 Jun;38(3):1339–1347. doi: 10.1016/j.clnu.2018.05.026
13. Muller AF, Fullwood L, Hawkins M, Cowley AJ. The Integrated Response of the Cardiovascular System to Food. Digestion 1992;52(3–4):184–193. doi: 10.1159/000200952
14. Niizeki K, Saitoh T. Analysis of cardiorespiratory phase coupling and cardiovascular autonomic responses during food ingestion. Physiol Behav 2016 May;159:1–13. doi: 10.1016/j.physbeh.2016.03.004
15. Ohnuki K, Niwa S, Maeda S, Inoue N, Yazawa S, Fushiki T. CH-19 Sweet, a Non-Pungent Cultivar of Red Pepper, Increased Body Temperature and Oxygen Consumption in Humans. Biosci Biotechnol Biochem 2001 Jan;65(9):2033–2036. doi: 10.1271/bbb.65.2033
16. Roth GM, Sheard C. MAINTENANCE OF VASODILATATION OF THE EXTREMITIES OF NORMAL INDIVIDUALS

FOR A PROLONGED PERIOD BY THE INGESTION OF TWO TO FOUR SUBSTANTIAL MEALS IN CLOSE SUCCESSION. Am J Physiol-Leg Content 1947 Dec 31;152(1):183–188. doi: 10.1152/ajplegacy.1947.152.1.183
17. Taylor JL, Curry TB, Matzek LJ, Joyner MJ, Casey DP. Acute Effects of a Mixed Meal on Arterial Stiffness and Central Hemodynamics in Healthy Adults. Am J Hypertens 2014 Mar 1;27(3):331–337. doi: 10.1093/ajh/hpt211
18. Waaler BA, Eriksen M. Post-prandial cardiovascular responses in man after ingestion of carbohydrate, protein or fat. Acta Physiol Scand 1992 Nov;146(3):321–327. doi: 10.1111/j.1748-1716.1992.tb09426.x
19. Waaler BA, Eriksen M, Janbu T. The effect of a meal on cardiac output in man at rest and during moderate exercise. Acta Physiol Scand 1990 Oct;140(2):167–173. doi: 10.1111/j.1748-1716.1990.tb08988.x
20. Abramson DI, Fierst SM. PERIPHERAL VASCULAR RESPONSES IN MAN DURING DIGESTION. Am J Physiol-Leg Content 1941 Jun 30;133(3):686–693. doi: 10.1152/ajplegacy.1941.133.3.686
21. Bagatell CJ, Heymsfield SB. Effect of meal size on myocardial oxygen requirements: implications for postmyocardial infarction diet. Am J Clin Nutr 1984 Mar;39(3):421–426. doi: 10.1093/ajcn/39.3.421
22. Fagan TC, Sawyer PR, Gourley LA, Lee JT, Gaffney TE. Postprandial alterations in hemodynamics and blood pressure in normal subjects. Am J Cardiol 1986 Sep;58(7):636–641. doi: 10.1016/0002-9149(86)90291-2
23. Grollman A. Physiological variations in the cardiac output of man III. The effect of pulse rate, blood pressure, and oxygen consumption of man. Am J Physiol-Leg Content 1929 Jul 1;89(2):366–370. doi: 10.1152/ajplegacy.1929.89.2.366
24. Smith A, Leekam S, Ralph A, McNeill G. The influence of meal composition on post-lunch changes in performance efficiency and mood. Appetite 1988 Jun;10(3):195–203. doi: 10.1016/0195-6663(88)90012-8
25. Waaler BA, Eriksen M, Toska K. The effect of meal size on postprandial increase in cardiac output. Acta Physiol Scand 1991 May;142(1):33–39. doi: 10.1111/j.1748-1716.1991.tb09125.x
