# Supplementary material for: Central Hemodynamic and Thermoregulatory Responses to Food Intake as Potential Biomarkers for Eating Detection: Systematic Review
Source: Interact J Med Res. 2024 Sep 10;13:e52167. doi: 10.2196/52167 (PMC11422732; doi:10.2196/52167)
Supplement: Multimedia Appendix 3 [file ijmr_v13i1e52167_app3.pdf]

Multimedia Appendix 3

Study Quality and Publication Bias Assessment

To assess overall study quality and risk of bias, we developed a custom tool comprising four categories: high, medium, low, and very low. This assessment tool was based on the Cochrane assessment framework. Two reviewers independently evaluated the study quality and risk of bias for each study, with an adjudicator resolving any conflicts.

*The following questions were multiple choice with **High**, **Low**, or **Unsure** as answers:*

1. **Sequence Generation:** Describe the method used to generate the allocation sequence in sufficient detail to allow an assessment of whether it should produce comparable groups.
2. **Allocation Concealment:** Describe the method used to conceal the allocation sequence in sufficient detail to determine whether intervention allocations could have been foreseen in advance of, or during, enrolment.
3. **Blinding of Participants and Personnel:** Describe all measures used, if any, to blind study participants and personnel from knowledge of which intervention a participant received. Provide any information relating to whether the intended blinding was effective.
4. **Blinding of outcome assessment:** Describe all measures used, if any, to blind outcome assessors from knowledge of which intervention a participant received. Provide any information relating to whether the intended blinding was effective.
5. **Incomplete Data:** Describe the completeness of outcome data for each main outcome, including attrition and exclusions from the analysis. State whether attrition and exclusions were reported, the numbers in each intervention group (compared with total randomized participants), reasons for attrition/exclusions where reported, and any re-inclusions in analyses performed by the review authors.
6. **Selective Reporting:** State how the possibility of selective outcome reporting was examined by the review authors, and what was found.
7. **Other Sources of Bias:** State any important concerns about bias not addressed in the other domains in the tool. If particular questions/entries were pre-specified in the review's protocol, responses should be provided for each question/entry.

*The following questions were multiple choice with **Yes**, **No**, or **Somewhat** as answers:*

8. Was the study objective or the research question clearly stated?
9. Was the study population clearly defined, specified, and representative of the general population of healthy individuals?
10. Was the data collected in a way that supports the research question or study objective?
11. Did the study describe the device(s) used to get physiological measurements?
12. Did the study describe the sensor resolution?
13. Were the results valid?
14. Were the results well described?

*To examine the potential of **publication bias**, we looked at each study's funding source, authors' conflicts of interest, and any other factors that could result in publication bias. We then rated each study into any of the three categories:*

- Undetected
- Strongly Suspected
- Very Strongly Suspected

*For the **overall study quality**, we classified each study into either of the four quality categories depending on the number of YES votes it received from two of the reviewers resulting in the following:*

- High Quality (13-14 YESs)
- Moderate Quality (11-12 YESs)
- Low Quality (9-10 YESs)
- Very Low Quality (<9 YESs)

If publication bias is undetected, the overall study quality is not affected; however, when it is strongly suspected, the overall study quality degrades by one level, and when it is very strongly suspected, the overall study quality degrades by two levels.

We present the study quality and publication bias for each of the included studies in Tables 10-18. Headings 1-9 represent answers for questions 1-9 above.

KEY

**Answer**	
Yes/Low	🟢
No/High	🔴
Somewhat/Unsure	🟡

Study	1	2	3	4	5	6	7	8	9	10	11	12	13	14	Publication Bias	Overall study Quality
[1]	🟢	🟢	🟡	🟢	🟢	🟢	🟢	🟢	🟡	🟢	🟡	🔴	🟢	🟢	Undetected	Low Quality
[2]	🟢	🟢	🟢	🟢	🟢	🟢	🟢	🟢	🟡	🟢	🟡	🔴	🟢	🟢	Undetected	Moderate Quality
[3]	🟢	🟢	🟢	🟢	🟢	🟢	🟢	🟢	🟡	🟢	🟡	🔴	🟢	🟢	Undetected	Moderate Quality
[4]	🟢	🟢	🟢	🟢	🟢	🟢	🟢	🟢	🟡	🟢	🟡	🔴	🟢	🟢	Undetected	Moderate Quality
[5]	🟢	🟢	🟢	🟢	🟢	🟢	🟢	🟢	🟢	🟢	🟢	🟡	🟢	🟢	Undetected	High Quality
[6]	🟢	🟢	🟢	🟢	🟢	🟢	🟢	🟢	🟢	🟢	🟢	🟡	🟢	🟢	Undetected	High Quality
[7]	🟢	🟢	🟢	🟢	🟢	🟢	🟢	🟢	🟢	🟢	🟢	🟡	🟢	🟢	Undetected	High Quality
[8]	🟢	🟢	🟢	🟢	🟢	🟢	🟢	🟢	🟡	🟢	🟡	🟡	🟢	🟢	Undetected	Moderate Quality
[9]	🟢	🟢	🟢	🟢	🟢	🟢	🟢	🟢	🟡	🟡	🟢	🟡	🟢	🟢	Undetected	Moderate Quality
[10]	🟢	🟢	🟢	🟢	🟢	🟢	🟢	🟢	🟡	🟢	🟡	🔴	🟢	🟢	Undetected	Moderate Quality
[11]	🟢	🟢	🟢	🟢	🟢	🟢	🟢	🟢	🟡	🟢	🔴	🔴	🟢	🟡	Undetected	Low Quality
[12]	🟢	🟢	🟢	🟢	🟢	🟢	🟢	🟢	🟢	🟢	🟢	🟡	🟢	🟢	Undetected	High Quality
[13]	🟢	🟢	🟢	🟢	🟢	🟢	🟢	🟢	🟢	🟢	🟢	🟢	🟡	🟢	Undetected	High Quality
[14]	🟢	🟢	🟢	🟢	🟢	🟢	🟢	🟢	🟡	🟢	🟢	🟢	🟢	🟢	Undetected	High Quality
[15]	🟢	🟢	🟢	🟢	🟢	🟢	🔴	🟢	🟡	🟡	🟢	🟡	🟢	🟢	Undetected	Low Quality
[16]	🟢	🟢	🟢	🟢	🟢	🟢	🟢	🟢	🟡	🟢	🟡	🔴	🟢	🟢	Undetected	Moderate Quality
[17]	🟢	🟢	🟢	🟢	🟢	🟢	🟢	🟢	🟢	🟢	🟢	🟢	🟢	🟢	Undetected	High Quality
[18]	🟢	🟢	🟢	🟢	🟢	🟢	🟢	🟢	🟡	🟢	🟢	🟢	🟢	🟢	Undetected	High Quality
[19]	🟢	🟢	🟢	🟢	🔴	🟢	🟢	🟢	🟡	🟢	🟢	🟡	🟢	🟡	Undetected	Low Quality
[20]	🟢	🟢	🟢	🟢	🟢	🟢	🟢	🟢	🟡	🟢	🟡	🔴	🟢	🟢	Undetected	Moderate Quality
[21]	🟢	🟢	🟢	🟢	🟢	🟢	🟢	🟢	🟡	🟢	🟡	🟡	🟢	🟢	Undetected	Moderate Quality

[22]	🟢	🟢	🟢	🟢	🟢	🟢	🟢	🟢	🟡	🟢	🟡	🟡	🟢	🟢	Undetected	Moderate Quality
[23]	🟢	🟢	🟢	🟢	🟢	🟢	🟢	🟢	🟢	🟢	🔴	🔴	🟢	🟡	Undetected	Moderate Quality
[24]	🟢	🟢	🟢	🟢	🟢	🟢	🟢	🟢	🟡	🟢	🔴	🔴	🟢	🟢	Undetected	Moderate Quality
[25]	🟢	🟢	🟢	🟢	🟢	🟢	🟢	🟢	🟡	🟢	🟢	🟡	🟢	🟢	Undetected	Moderate Quality

References

1. Dagenais GR, Oriol A, McGregor M. Hemodynamic effects of carbohydrate and protein meals in man: rest and exercise. J Appl Physiol 1966 Jul;21(4):1157–1162. doi: 10.1152/jappl.1966.21.4.1157
2. De Mey C, Enterling D, Brendel E, Meineke I. Postprandial changes in supine and erect heart rate, systemic blood pressure and plasma noradrenaline and renin activity in normal subjects. Eur J Clin Pharmacol 1987 Sep;32(5):471–476. doi: 10.1007/BF00637672
3. De Mey C, Hansen-Schmidt S, Enterling D. Postprandial haemodynamic changes: a source of bias in cardiovascular research affected by its own methodological bias. Cardiovasc Res 1988 Oct 1;22(10):703–707. doi: 10.1093/cvr/22.10.703
4. De Mey C, Hansen-Schmidt S, Enterling D, Meineke I. Time course and nature of postprandial haemodynamic changes in normal man. Clin Physiol 1989 Feb;9(1):77–87. doi: 10.1111/j.1475-097X.1989.tb00958.x
5. Dencker M, Björgell O, Hlebowicz J. Effect of Food Intake on Commonly Used Pulsed Doppler and Tissue Doppler Measurements: Food Intake and Doppler Measurements. Echocardiography 2011 Sep;28(8):843–847. doi: 10.1111/j.1540-8175.2011.01451.x
6. Hauser JA, Muthurangu V, Steeden JA, Taylor AM, Jones A. Comprehensive assessment of the global and regional vascular responses to food ingestion in humans using novel rapid MRI. Am J Physiol-Regul Integr Comp Physiol 2016 Mar 15;310(6):R541–R545. doi: 10.1152/ajpregu.00454.2015
7. Hawley SK, Channer KS. Relative effects of fat-, carbohydrate- and protein-containing liquid diets on cardiac output in healthy adult subjects. Clin Sci 1992 Oct 1;83(4):483–487. doi: 10.1042/cs0830483
8. Høst U, Kelbaek H, Rasmusen H, Court-Payen M, Christensen NJ, Pedersen-Bjergaard U, Lorenzen T. Haemodynamic Effects of Eating: The Role of Meal Composition. Clin Sci 1996 Apr 1;90(4):269–276. doi: 10.1042/cs0900269
9. Ishimine M, Takamoto T, Nitta M, Marumo F, Murakami K, Takasu N. Postprandial Hemodynamic Changes Evaluated by a Doppler Echocardiographic Method. Jpn Heart J 1994;35(1):35–42. doi: 10.1536/ihj.35.35
10. Kelbaek H, Munck O, Christensen NJ, Godtfredsen J. Central haemodynamic changes after a meal. Heart 1989 Jun 1;61(6):506–509. doi: 10.1136/hrt.61.6.506
11. Macht MB, Pillion EL. Changes in skin temperature and blood flow of hand following ingestion of certain amino acids. Fed Proc 1948 Mar;7(1 Pt):75. PMID:18938593
12. Martinez-Tellez B, Ortiz-Alvarez L, Sanchez-Delgado G, Xu H, Acosta FM, Merchan-Ramirez E, Muñoz-Hernandez V, Martinez-Avila WD, Contreras-Gomez MA, Gil A, Labayen I, Ruiz JR. Skin temperature response to a liquid meal intake is different in men than in women. Clin Nutr 2019 Jun;38(3):1339–1347. doi: 10.1016/j.clnu.2018.05.026
13. Muller AF, Fullwood L, Hawkins M, Cowley AJ. The Integrated Response of the Cardiovascular System to Food. Digestion 1992;52(3–4):184–193. doi: 10.1159/000200952
14. Niizeki K, Saitoh T. Analysis of cardiorespiratory phase coupling and cardiovascular autonomic responses during food ingestion. Physiol Behav 2016 May;159:1–13. doi: 10.1016/j.physbeh.2016.03.004
15. Ohnuki K, Niwa S, Maeda S, Inoue N, Yazawa S, Fushiki T. CH-19 Sweet, a Non-Pungent Cultivar of Red Pepper, Increased Body Temperature and Oxygen Consumption in Humans. Biosci Biotechnol Biochem 2001 Jan;65(9):2033–2036. doi: 10.1271/bbb.65.2033
16. Roth GM, Sheard C. MAINTENANCE OF VASODILATATION OF THE EXTREMITIES OF NORMAL INDIVIDUALS FOR A PROLONGED PERIOD BY THE INGESTION OF TWO TO FOUR SUBSTANTIAL MEALS IN CLOSE SUCCESSION. Am J Physiol-Leg Content 1947 Dec 31;152(1):183–188. doi: 10.1152/ajplegacy.1947.152.1.183
17. Taylor JL, Curry TB, Matzek LJ, Joyner MJ, Casey DP. Acute Effects of a Mixed Meal on Arterial Stiffness and Central Hemodynamics in Healthy Adults. Am J Hypertens 2014 Mar 1;27(3):331–337. doi: 10.1093/ajh/hpt211

18. Waaler BA, Eriksen M. Post-prandial cardiovascular responses in man after ingestion of carbohydrate, protein or fat. Acta Physiol Scand 1992 Nov;146(3):321–327. doi: 10.1111/j.1748-1716.1992.tb09426.x
19. Waaler BA, Eriksen M, Janbu T. The effect of a meal on cardiac output in man at rest and during moderate exercise. Acta Physiol Scand 1990 Oct;140(2):167–173. doi: 10.1111/j.1748-1716.1990.tb08988.x
20. Abramson DI, Fierst SM. PERIPHERAL VASCULAR RESPONSES IN MAN DURING DIGESTION. Am J Physiol-Leg Content 1941 Jun 30;133(3):686–693. doi: 10.1152/ajplegacy.1941.133.3.686
21. Bagatell CJ, Heymsfield SB. Effect of meal size on myocardial oxygen requirements: implications for postmyocardial infarction diet. Am J Clin Nutr 1984 Mar;39(3):421–426. doi: 10.1093/ajcn/39.3.421
22. Fagan TC, Sawyer PR, Gourley LA, Lee JT, Gaffney TE. Postprandial alterations in hemodynamics and blood pressure in normal subjects. Am J Cardiol 1986 Sep;58(7):636–641. doi: 10.1016/0002-9149(86)90291-2
23. Grollman A. Physiological variations in the cardiac output of man III. The effect of pulse rate, blood pressure, and oxygen consumption of man. Am J Physiol-Leg Content 1929 Jul 1;89(2):366–370. doi: 10.1152/ajplegacy.1929.89.2.366
24. Smith A, Leekam S, Ralph A, McNeill G. The influence of meal composition on post-lunch changes in performance efficiency and mood. Appetite 1988 Jun;10(3):195–203. doi: 10.1016/0195-6663(88)90012-8
25. Waaler BA, Eriksen M, Toska K. The effect of meal size on postprandial increase in cardiac output. Acta Physiol Scand 1991 May;142(1):33–39. doi: 10.1111/j.1748-1716.1991.tb09125.x
