# Supplementary material for: Central Hemodynamic and Thermoregulatory Responses to Food Intake as Potential Biomarkers for Eating Detection: Systematic Review
Source: Interact J Med Res. 2024 Sep 10;13:e52167. doi: 10.2196/52167 (PMC11422732; doi:10.2196/52167)
Supplement: Multimedia Appendix 4 [file ijmr_v13i1e52167_app4.pdf]

Multimedia Appendix 4

Post-prandial physiological responses and the number of studies reporting data for each category

Physiological Response	Post-prandial increase	Post-prandial decrease	Inconsistent response	Statistically insignificant response
Heart Rate	19			
Systolic Blood Pressure	7	1		4
Diastolic Blood Pressure	1	8		3
Mean Blood Pressure			1	3
Oxygen Uptake	3		1	1
Cardiac Output	18			1
Stroke Volume	11			
Systemic Vascular Resistance		2		
Superior Mesenteric Vascular Resistance		1		
Hand Blood Flow	1			2
Calf Blood Flow	1			1
Superior Mesenteric Blood Flow	1			
Proximal and distal skin temperature	2		4	1
Core Temperature			1	
